# Vascular Neurology Nurse Practitioner Provision of Telemedicine Consultations

**DOI:** 10.1155/2010/507071

**Published:** 2010-08-02

**Authors:** Bart M. Demaerschalk, Terri-Ellen J. Kiernan, STARR Investigators

**Affiliations:** Division of Cerebrovascular Diseases and Division of Critical Care Neurology, Department of Neurology, Mayo Clinic Hospital, 5777 East Mayo Boulevard, Phoenix, AZ 85054, USA

## Abstract

*Objective*. The objective was to define and evaluate a role for the Vascular Neurology-Nurse Practitioner (VN-NP) in the delivery of telemedicine consultations in partnership with a vascular neurologist. *Methods*. Prospective stroke alert patients at participating hospitals underwent a two-way audio video telemedicine consultation with a VN-NP at a remotely located stroke center in partnership with a vascular neurologist. Demographic information, National Institutes of Health Stroke Scale (NIHSS) scores, diagnoses, CT contraindications to thrombolysis, thrombolysis eligibility, and time interval data were collected. The inter-rater agreement between VN-NP and vascular neurologist assessments was calculated. *Results*. Ten patients were evaluated. Four were determined to have ischemic stroke, one had a transient ischemic attack, two had intracerebral hemorrhages, and three were stroke mimics. Overall, three patients received thrombolysis. The inter-rater agreement between VN-NP and vascular neurologist assessments were excellent, ranging from 0.9 to 1.0. The duration of VN-NP consultation was 53.2 ± 9.0 minutes, which included the vascular neurologist supervisory evaluation time of 12.0 ± 9.6 minutes. *Conclusion*. This study illustrated that a stroke center VN-NP, in partnership with a vascular neurologist, could deliver timely telemedicine consultations, accurate diagnoses, and correct treatments in acute stroke patients who presented to remotely located rural emergency departments within a hub and spoke network. VN-NPs may fulfill the role of a telestroke provider.

## 1. Introduction

The purpose of this preliminary study was to define, demonstrate, and evaluate a role for the vascular neurology nurse practitioner (VN-NP) in the delivery of telemedicine consultations in partnership with a vascular neurologist in the context of an established hub and spoke stroke telemedicine network. NPs, physician assistants (PAs), and other physician extenders are no longer being relegated to subservient roles in health-care delivery. They are assuming an ever-increasing level of responsibility in patient care. With technological enablers, such as telemedicine, physician extenders' future roles as specialty caregivers in rural communities will grow. Examples of physician—NP telemedicine partnerships already exist in rural emergency medicine, but none describe the potential role of a VN-NP telemedicine provider responding to cases of acute stroke. 

Physician extenders and midlevel providers have practiced emergency medicine for 25 years or more [1]. In the United States, it is estimated that NP and PA are involved in 1.8% and 6.5%, respectively, of emergency department consultations [2]. Nearly half of all emergency departments employ midlevel providers [3]. Historically, emergency medicine midlevel providers were positioned in the hospital; when or if, supervision was necessary, emergency physicians and/or medical or surgical specialists were consulted. With telemedicine, an NP positioned in an emergency department could consult a remotely located physician. For example, in the published program, “TelEmergency for rural hospitals,” emergency NP collaborated with remote TelEmergency physicians to treat patients [4]. The NP was required to have specific qualifications, including master's degree, certification as a family NP with an unrestricted license, and state license eligibility [4]. In the TelEmergency system, the cost of 24/7 NP and TelEmergency physician staffing partnership was US $53,000 per month per spoke, compared with an estimated US $72,000 per month for a traditional physician staffing model [4]. The NP and physician partnership system allowed participating spoke hospitals to provide emergency services equivalent to a physician-only model while realizing significant cost savings. In TelEmergency, stroke presentation was a top-ten most common complaint category in the patients over 75 years of age, representing 6% of the diagnoses. Overall patient satisfaction with a TelEmergency program was very high, 94% indicating comfort in the system and practitioner partnership [4]. Other countries, including Australia, England, Scotland, Netherlands, and Canada have adopted midlevel providers in rural and remote regions to address workforce shortage [5]. The midlevel providers have provided safe, high-quality, and cost-effective care. There are many published examples of both NPs and PAs practicing telemedicine [6–9]. 

In vascular neurology, it is notable that there is a rural metropolitan disparity in acute stroke care with a shortage of vascular neurologists and an aging-aware population. There is more public pressure than ever before. Healthcare organizations are answering by establishing primary stroke centers. However, acquiring the needed work force remains challenging. One solution would be to utilize specialized NPs or PAs. 

The primary objective of this preliminary study was to establish the feasibility of a VN-NP and a supervising vascular neurologist partnership to respond, emergently, to telestroke hotline activations in a single hub, multirural spoke hospital telestroke state network. A secondary objective was to assess agreement between VN-NP and vascular neurologist over the NIHSS score, diagnosis (stroke or nonstroke), head CT interpretation (radiological contraindication to thrombolysis or not), and overall thrombolysis eligibility (yes or no). Thirdly, the encounter time (minutes) intervals of the VN-NP vascular neurologist consultative partnership experience were evaluated.

## 2. Methods

A fully operational single-hub, multirural spoke hospital telestroke network existed in Arizona, United States of America. A description of how the early hub and spoke network was established with descriptions of the technological factors, information technology, security, data encryption, the audio visual (AV) camera system, and technique, and prospective reliability have already been published [9–14]. 

The hub stroke team (on-call 24 hours per day, 7 days per week) was contacted directly by a ring-central operated alphanumeric group pager system or smart phone when a patient with acute stroke symptoms presented at the spoke emergency department. When on-call with the stroke team (1 week per month) the designated hub VN-NP telephoned the applicable spoke emergency department and spoke briefly with the spoke emergency physician in order to determine patient eligibility status for AV consultation. The published STRokE DOC AZ TIME and STARR AV telestroke consultation algorithm was replicated [10, 15]. Eligible consented patients underwent consultation. The hub VN-NP established audio and video contact with the spoke site and immediately acquired a medical history from patients and all the accompanying relatives, supplemented by verbal and written reports from emergency medical systems (EMS), physicians, and nursing staff. 

Following the history acquisition, the VN-NP, certified in National Institutes of Health stroke scale (NIHSS) examination, performed the evaluation with the aid of healthcare provider staff at the spoke site. Other relevant elements of the examination were performed by, or reported to, the NP as appropriate. Diagnostic test results were reported to the VN-NP by the spoke emergency physician, either verbally or by electronic-fax (e-fax). The e-fax is a system in which faxed material is received via e-mail. Head computed tomography (CT) images were viewed by the hub VN-NP with digital imaging and communications in medicine (DICOM) viewer. The hub VN-NP completed a prespecified consultation report form, which included acute time intervals, eligibility criteria checklist for thrombolysis, NIHSS scoring, CT evaluation checklist, and laboratory findings. Clinical deficit and functional scale scores (including the NIHSS and prestrike- and poststroke- modified Rankin scale (mRS) score) were calculated by the VN-NP with the information provided by the bedside emergency physician or other healthcare providers. After a review of the history, the examination findings, stroke scales, head CT interpretation, laboratory results, and electrocardiogram, the VN-NP communicated with the supervising hub vascular neurology consultant.

Communication between the VN-NP and vascular neurologist was generally by telephone as they were not in the same location. In every consultation the VN-NP presented a synthesis of the case, the diagnosis, and a recommendation regarding patient eligibility for intravenous thrombolysis to the supervising consultant. The consultant, also certified in the NIHSS examination, established audio and video contact with the spoke site and was free to repeat, or request a repeated, examination item and could interact with patient, relatives, witnesses, and emergency nurses and review head CT. Within approximately 10 minutes, the consultant notified the VN-NP of whether or not there was agreement regarding the NIHSS score, CT interpretation, diagnosis, and treatment recommendation. Once consensus was reached (within approximately 5 minutes), the VN-NP presented the recommendation regarding patient diagnosis and eligibility for intravenous thrombolysis to the spoke emergency physician. The VN-NP dictated a consultation summary note, and the consultant added a brief supervisory note. Once transcribed, both were transmitted to spoke emergency department by e-fax. Copies were maintained in the hub and spoke healthcare records.

Hub hospital stroke center providers included a Neurovascular Education and Training in Stroke Management and Acute Reperfusion Therapy (NET SMART) NP graduate and five vascular neurologists. Equipment included internet-enabled desktops and laptops with cameras for hub providers and telemedicine platform systems at remote emergency departments [16]. The software enabled site-independent access to two-way audio and high-resolution video, over standard internet connections (BF Technologies, San Diego, CA, USA).

Data from ten prospective VN-NP telestroke consultations, supervised by a vascular neurologist, were collected during the interval from November 2008 to November 2009. The study was approved by each of the participating spoke hospital institutional review boards (IRB) and also by Mayo Clinic IRB, with authorization for central oversight.

## 3. Statistical Analysis

This preliminary paper was principally a feasibility study. The study sample size was 10 cases. Analyses included mean and standard deviation of time categories and kappa coefficient for inter rater agreement.

## 4. Results

Table 1 displays basic demographic information, severity of deficit, diagnosis, CT observation, and thrombolysis eligibility of this study cohort. The VN-NP telestroke consultation patients did not differ substantially, in characteristics, from those patients in similar trials [10, 12]. The VN-NP telestroke consultation patients were broadly representative of typical patients seen by emergency stroke teams, for example, similar proportions of ischemic and hemorrhagic stroke, transient ischemic attack, and stroke mimics. Thirty percent of the VN-NP consultation patients were determined to have ischemic stroke and were eligible for thrombolysis, similar to the proportion in other telestroke trials [10, 12]. The inter rater agreement between VN-NP and vascular neurologist (Table 2) was excellent for NIHSS score, diagnosis, head CT interpretation, and overall thrombolysis eligibility.

## 5. Discussion

There are many facets of this small study worthy of discussion, including: comparing time intervals between telemedicine studies, feasibility, scope of practice of mid level providers, neurovascular specialty training, and future considerations. 

 The time intervals of the VN-NP vascular neurologist consultative partnership (Table 3) were similar to those of previously published traditional vascular neurologist service provision in telestroke trials in the same network (STRokE DOC AZ) [7] and a comparable network in a neighboring state (STRokE DOC) [12]. Compared to physician-only telestroke consultations, the VN-NP algorithm resulted in approximately 10 minutes faster call-to-neurology exam and 10 to 20 minutes faster consent-to-neurology exam intervals, but 20 to 30 minutes slower decision-to-rt-PA interval. The VN-NP appeared to respond quickly to telestroke alert calls from spoke hospitals, progressing swiftly from the emergency alert to starting the consultation. On the other hand, the decision (for tPA eligibility) to tPA administration was relatively long, 45 minutes, in this study. The result represents only three (of 10 total) thrombolysed subjects. The sample was very small. Events needing to occur between VN-NP decision and thrombolysis include: supervising vascular neurologist evaluation, consensus, communication with emergency physician, communication with patient/family, pharmacy order for tPA, drug preparation, and then administration. Occasionally elevated blood pressure required treatment before tPA was administered. Despite the added complexity of two neurology providers assessing each patient and communicating with one another, the overall time intervals of call-to-decision and consent-to-rt-PA were similar to intervals reported by STRokE DOC trials [12, 15]. The supervising vascular neurologists required an average 12.0 minutes to assess each shared telestroke case, in contrast to an average requirement of 58.3 to 64.7 minutes per case without VN-NP partnership [12, 15]. Even the cumulative total of VN-NP plus vascular neurologist time requirement (65.2 minutes) was not substantially different from the published time required by a solo vascular neurologist (58.3 to 64.7 minutes) [12, 15]. 

The results from this preliminary study demonstrated the feasibility of establishing a VN-NP and a supervising vascular neurologist partnership to respond, emergently, to telestroke hotline activations in a single hub, multirural spoke hospital telestroke state network compared to those of published traditional vascular neurology consultant telestroke service provision in the same network (STRokE DOC AZ) and a comparable network in a neighboring state (STRokE DOC) [12, 15]. This bodes well in a relatively busy telemedicine network hub responding to many, and sometimes, simultaneous stroke alerts. 

 The algorithm proposed is built upon the success of midlevel providers practicing emergency medicine and telemedicine, but there is a fundamental difference. In our study algorithm, both the VN-NP and the vascular neurology physician specialist were practicing telemedicine remote from the emergency patient. The concept of telestroke is already a decade old but its broad and growing utilization is relatively novel. Elements of this consultative modality remain controversial, debated, researched and pose limitations and obstacles to wide-scale adoption. We believe this to be the first published description of VN-NPs as telestroke providers. We anticipate the continued, and growing, need for stroke providers and suspect that vascular neurologist numbers, alone, will not suffice. Stroke-trained midlevel practitioners, together with telemedicine, may provide a practical and cost-effective solution. 

In this study, it is important to clarify the scope of practice for NPs and PAs. The healthcare organization, Mayo Clinic, where this study took place, spans three states and employs both NPs and PAs. The policies regulating the scope of practice for NPs and PAs are consistent system wide and are collectively classified as Advanced Midlevel Practitioners (AMLPs). Many healthcare organizations have policies governing the practice of AMLPs that may differ from the state boards.

A study funded by the National Center for Health Workforce Analysis bureau of Health Professions compared changes in the professional practice for NPs, PAs, and certified nurse midwives from 1992 and 2000 [17]. The PA is a physician extender whose scope of practice is determined by the supervising physician who delegates or assigns duties within their specialty. However state laws and regulations play a significant role as well [18] whereas in most states NPs are not dependent on upon physician delegation or supervision with autonomy and have full prescriptive rights [19].

In this study the supervision of the NP by the collaborating physician is important. Stroke telemedicine is a new frontier. Recently, vascular neurologists were conducting studies to determine if they could accurately and safely make decisions on the use of thrombolysis for acute stroke utilizing audio-video consultation. The research team determined that a partnership of a physician and NP would be a conservative and safe next step for a pilot study. The results could influence the development of collaborative or solo NP acute stroke telemedicine practices.

Many NPs and PAs are becoming more subspecialized in their practice. The NET SMART-APN is the first-ever fellowship for neurovascular care. It is a federally funded, evidence-based, neurovascular fellowship program supported by the Healthcare Services and Resources Administration. Eighty-one APNs are currently enrolled in the curriculum, and 15 will graduate in 2010. At stroke centers with newly graduated NET SMART APN fellows, the thrombolysis treatment rates have increased substantially [16]. The NET SMART program includes 14 learning modules, with “CT Imaging in Acute Stroke” included as a fundamental topic. The 28-hour module incorporated both online didactic and preceptored training by vascular neurologists and Neuroradiologists with 20 acute stroke cases to be signed off for accuracy. NET SMART graduates who become proficient with telemedicine techniques have all the requisite skills to serve as providers for rurally located stroke patients in the emergency department, the hospital, and during postdischarge followup clinics. 

Acute care NPs role in telemedicine practice is expanding. According to the Joint Commission, the medical staff determines which services can be appropriately delivered via telemedicine [20]. All practitioners, including NPs, who diagnose and treat patients using telemedicine technology, are subject to the credentialing and privileging processes of the receiving organization [20]. A receiving organization may elect to use the credentialing information gathered by another JC accredited facility, provided that the receiving organization makes the decisions delineating privileges [20]. The Arizona Board of Nursing does not make reference to NPs and telemedicine. NPs do not require a supervising physician for treating patients in Arizona although they are expected to consult a physician whenever they believe the situation warrants it.

## 6. Conclusion

This preliminary study was limited, principally by size (10 consultations) and the lack of a direct comparison arm. Nonetheless, the study was the first to demonstrate that a VN-NP, in partnership with a vascular neurologist, could deliver timely telemedicine consultations, accurate diagnoses, and correct treatments in acute stroke patients who presented to rural emergency departments within an established hub and spoke network. Future larger studies on this topic should be designed to compare timeliness, accuracy, decision making, effectiveness, short and long term clinical outcomes, and cost of the VN-NP versus vascular neurologist-only model for acute stroke care in rural community settings. This VN-NP and a telemedicine practice partnership with vascular neurologists may be viable answers to the critical rural-urban disparity of acute stroke management practices.

## Figures and Tables

**Table 1:** Demographic information, diagnoses, and thrombolysis eligibility.

	VN-NP Telemedicine Algorithm (*N* = 10)
Gender (% Female)	50.0
Age (Mean, Years)	70.8
NIHSS Score (Mean)	11.6
Ischemic Stroke (%)	40.0
Intracerebral Hemorrhage (%)	20.0
TIA (%)	10.0
Stroke Mimic (%)	30.0
CT contraindication to thrombolysis (%)	20.0
Thrombolysis Administered (proportion of consultations)	30.0

**Table 2:** VN-NP and Vascular Neurologist Inter rater Agreement.

Assessment	Kappa (95% CI, if applicable)
NIHSS Score	0.859 (0.734 to 0.984)
Stroke Diagnosis	1.0
CT contraindication to thrombolysis	1.0
Thrombolysis eligibility	1.0

**Table 3:** Consultation time intervals.

Time Interval	VN-NP Telemedicine Algorithm (mean and standard deviation, minutes)
Onset to Door	32.3 ± 20.5
Onset to Call	42.8 ± 21.2
Onset to Lab	110.5 ± 12.0
Onset to Decision	112.6 ± 31.1
Onset to rt-PA	159.0 ± 8.5
Door to Call	19.6 ± 18.7
Door to Consent	39.8 ± 26.0
Door to Lab	75.0 ± 32.4
Door to Neuro Exam	54.2 ± 15.1
Door to CT Reading	78.2 ± 23.4
Door to Decision	69.6 ± 9.6
Call to Consent	21.0 ± 10.8
Call to Neuro Exam	31.5 ± 11.5
Call to Decision	53.2 ± 9.0
Consent to Neuro Exam	9.0 ± 16.8
Consent to Decision	30.7 ± 19.3
Consent to rt-PA	67.0 ± 30.5
Decision to rt-PA	45.5 ± 21.9

Definitions: Onset:stroke symptom onset time or the time the subject was last known to be at baseline state; Door:emergency department triage time; Lab:time lab results reviewed; Decision:time that thrombolysis eligibility was determined; rt-PA:time of IV rt-PA administration; Consent:time of subject or representative written consent; Neuro Exam:time that the NIHSS evaluation started; CT Reading:time that CT was interpreted.

## References

[1] US Congress, Office of Technology Assessment (1986). *Nurse Practitioners, Physician Assistants, and Certified Nurse-Midwives: A Policy Analysis*.

[2] Centers for Disease Control and Prevention National Hospital Ambulatory Medical Survey: 2003 Emergency Department Summary.

[3] Hooker RS, McCaig L (1996). Emergency department uses of physician assistants and nurse practitioners: a national survey. *American Journal of Emergency Medicine*.

[4] Galli R, Keith JC, McKenzie K, Hall GS, Henderson K (2008). TelEmergency: a novel system for delivering emergency care to rural hospitals. *Annals of Emergency Medicine*.

[5] O’Connor TM, Hooker RS (2007). Extending rural and remote medicine with a new type of health worker: physician assistants. *Australian Journal of Rural Health*.

[6] Staller JA (2006). Psychiatric nurse practitioners in rural pediatric telepsychiatry. *Psychiatric Services*.

[7] Armer JM (2003). A case study of the use of telemedicine by advanced practice nurses in rural Missouri. *The Journal of Continuing Education in Nursing*.

[8] Asprey DP, Zollo S, Kienzle M (2001). Implementation and evaluation of a telemedicine course for physician assistants. *Academic Medicine*.

[9] Goldstein N (2005). Look what’s next in telemedicine: the physician assistant and telemedicine. *Hawaii Medical Journal*.

[10] Demaerschalk BM, Miley ML, Kiernan T-EJ (2009). Stroke telemedicine. *Mayo Clinic Proceedings*.

[11] Miley ML, Demaerschalk BM, Olmstead NL (2009). The state of emergency stroke resources and care in rural arizona: a platform for telemedicine. *Telemedicine and e-Health*.

[12] Meyer BC, Raman R, Hemmen T (2008). Efficacy of site-independent telemedicine in the STRokE DOC trial: a randomised, blinded, prospective study. *The Lancet Neurology*.

[13] Meyer BC, Lyden PD, Al-Khoury L (2005). Prospective reliability of the STRokE DOC wireless/site independent telemedicine system. *Neurology*.

[14] Meyer BC, Raman R, Chacon MR, Jensen M, Werner JD (2008). Reliability of site-independent telemedicine when assessed by telemedicine-naïve stroke practitioners. *Journal of Stroke & Cerebrovascular Diseases*.

[15] Demaerschalk BM, Bobrow BJ, Raman R (2010). Stroke team remote evaluation using a digital observation camera in Arizona: the initial mayo clinic experience trial. *Stroke*.

[16] Alexandrov AW, Brethour M, Cudlip F (2009). Post-coraduate fellowship education and training for nurses: the NET SMART experience. *Critical Care Nursing Clinics of North America*.

[17] U.S. Department of Health and Human Services A comparison of changes in the professional practice of Nurse Practitioners, Physician Assistants, and Certified Nurse Midwives: 1992 and 2000. http://bhpr.hrsa.gov/healthworkforce/reports/scope/scope1-2.htm.

[18] American Academy of Physician Assistants http://aapa.org/advocacy-and-practice-resources/state-government-and-licensing/scope-of-practice.

[19] American College of Nurse Practitioners NP Scope of Practice. http://www.acnpweb.org/i4a/pages/index.cfm?pageid=3465.

[20] Magdic KS, Hravnak M, McCartney S (2005). Credentialing for nurse practitioners: an update. *AACN Clinical Issues*.

